# VDR activation attenuate cisplatin induced AKI by inhibiting ferroptosis

**DOI:** 10.1038/s41419-020-2256-z

**Published:** 2020-01-29

**Authors:** Zhaoxin Hu, Hao Zhang, Bin Yi, Shikun Yang, Jun Liu, Jing Hu, Jianwen Wang, Ke Cao, Wei Zhang

**Affiliations:** 1 Department of Nephrology, The Third Xiangya Hospital, Central South University, Changsha, 410013 Hunan Province China; 2 Department of Oncology, The Third Xiangya Hospital, Central South University, Changsha, 410013 Hunan Province China

**Keywords:** Cell death, Kidney diseases

## Abstract

Our preliminary work has revealed that vitamin D receptor (VDR) activation is protective against cisplatin induced acute kidney injury (AKI). Ferroptosis was recently reported to be involved in AKI. Here in this study, we investigated the internal relation between ferroptosis and the protective effect of VDR in cisplatin induced AKI. By using ferroptosis inhibitor ferrostatin-1 and measurement of ferroptotic cell death phenotype in both in vivo and in vitro cisplatin induced AKI model, we observed the decreased blood urea nitrogen, creatinine, and tissue injury by ferrostatin-1, hence validated the essential involvement of ferroptosis in cisplatin induced AKI. VDR agonist paricalcitol could both functionally and histologically attenuate cisplatin induced AKI by decreasing lipid peroxidation (featured phenotype of ferroptosis), biomarker 4-hydroxynonenal (4HNE), and malondialdehyde (MDA), while reversing glutathione peroxidase 4 (GPX4, key regulator of ferroptosis) downregulation. VDR knockout mouse exhibited much more ferroptotic cell death and worsen kidney injury than wild type mice. And VDR deficiency remarkably decreased the expression of GPX4 under cisplatin stress in both in vivo and in vitro, further luciferase reporter gene assay showed that GPX4 were target gene of transcription factor VDR. In addition, in vitro study showed that GPX4 inhibition by siRNA largely abolished the protective effect of paricalcitol against cisplatin induced tubular cell injury. Besides, pretreatment of paricalcitol could also alleviated Erastin (an inducer of ferroptosis) induced cell death in HK-2 cell. These data suggested that ferroptosis plays an important role in cisplatin induced AKI. VDR activation can protect against cisplatin induced renal injury by inhibiting ferroptosis partly via trans-regulation of GPX4.

## Introduction

AKI is a common and critical illness which occurs in approximately 5% of hospitalized patients and 30% of critically ill patients and has high morbidity and mortality^1^. Cisplatin is one of the major causes of clinical AKI, while the precise molecular mechanisms were not completely clear, very limited strategies for AKI prevention or therapy are available at hand^2^. Apoptosis and necroptosis were previously reported to be the major pathological processes of acute kidney injury, however, preclinical works using specific inhibitor of neither apoptosis or necroptosis failed to completely prevent or stop cisplatin induced AKI^3–5^. Indicating that other types of cell death may also at least co-exists in cisplatin induced AKI.

Ferroptosis is a type of “regulated cell death” recently identified as an iron- and lipid hydroperoxide-dependent nonapoptotic cell death in cancer cells^6^. Ferroptosis has been implicated in the pathological procedures of a lot of diseases like Huntington’s, carcinogenesis, stroke, intracerebral hemorrhage, ischemia-reperfusion injury of kidney, and liver^7^. However, whether ferroptosis are involved in cisplatin induced AKI (cis-AKI) were not clearly verified yet. Early in 1998 it was reported that exposure to cisplatin resulted in a significant increase in bleomycin-detectable iron in kidney cells and the use of deferoxamine significantly alleviated kidney injury induced by cisplatin in vivo^8^. Our pre-experiments showed that pretreatment of ferrostatin-1 (Fer-1), an inhibitor of ferroptosis, has significantly decreased the blood Urea Nitrogen (BUN) and serum creatinine(sCr) level in cis-AKI mice model.

Vitamin D receptor (VDR) is a member of the nuclear receptor superfamily which is activated by its ligand 1,25-dihydroxyvitamin D3 (1,25(OH)2D3) or other agonists like paricalcitol to exert its transcription regulatory effect^9^.

Numerous studies including our previous work have shown that 1,25(OH)2D3 or its active analogs, through VDR activation, are protective against proteinuria, renal fibrosis, and inflammation in patients with chronic kidney disease and in animal models of diabetic nephropathy or end-stage renal disease^10–13^. However, few attentions were focused on the role of VDR in AKI. Our preliminary data of this work has found that VDR agonist paricalcitol could also attenuate cis-AKI in a similar way as Fer-1. Thus, in the present study we first verified the involvement of ferroptosis in cis-AKI, and further investigated whether the VDR activation plays a protective role in cis-AKI through targeting ferroptotic cell death. Our data has validated the essential role of ferroptosis in the process of cis-AKI, and VDR signaling can protect against cisplatin induced renal injury partly by inhibiting ferroptosis via a transcription regulation mechanism.

## Results

### Ferroptosis is involved in cisplatin induced AKI

**Fig. 1:**
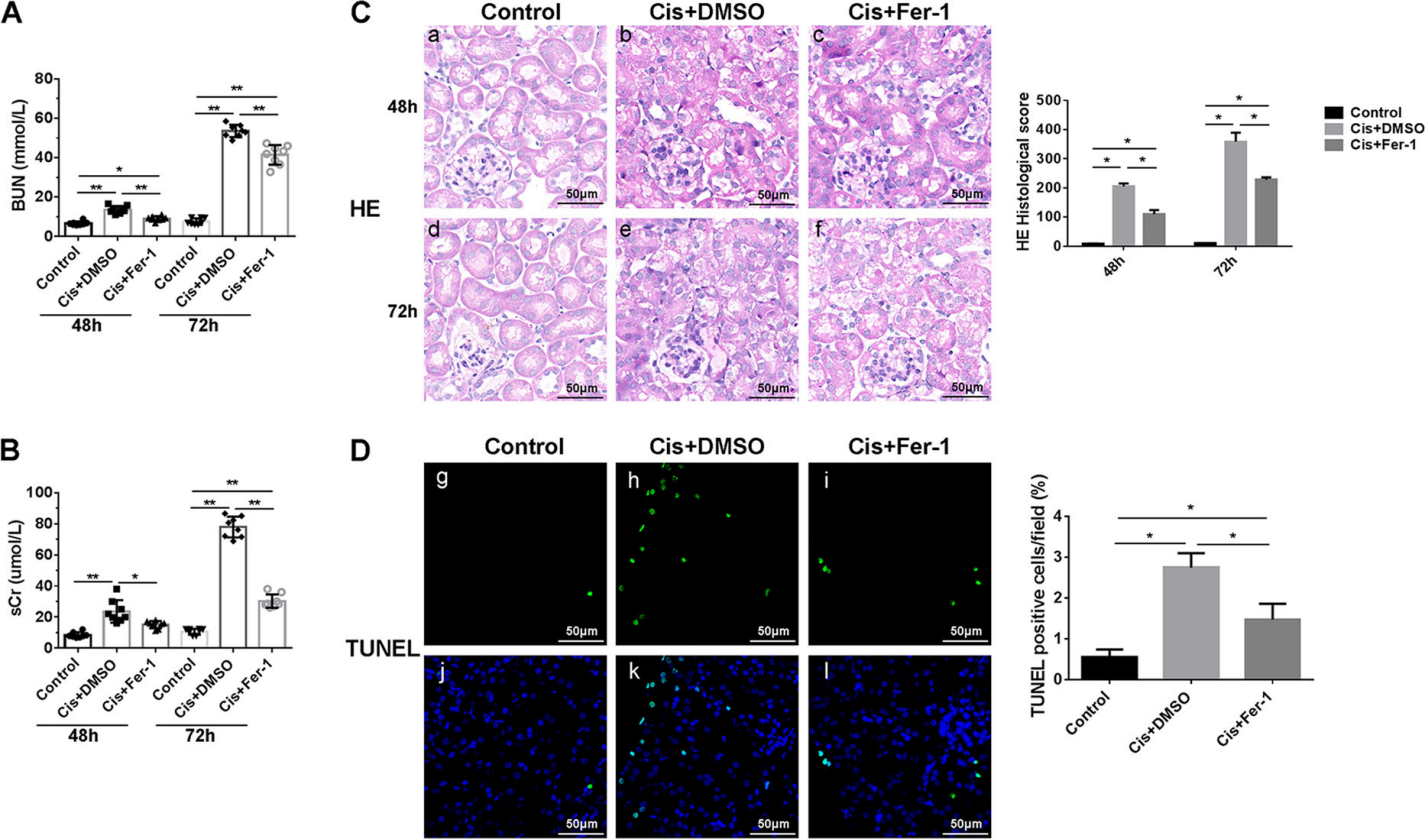
Cisplatin induced Renal injury were alleviated by Ferroptosis inhibitor Ferrostatin-1(Fer-1). **A**, **B** Serum BUN and Cr levels of each group at 48 h and 72 h after cisplatin injection. **C** HE staining at 48 h (**a–c**) and 72 h (**d–f**) after cisplatin and Fer-1 injection, and histological scores analysis of renal tubular damage in each group. Scale bar = 50 μm. **D** TUNEL fluorescence staining and statistical analysis of cell death rates in each group at 48 h after cisplatin treatment. Green fluorescence in pictures (**g–i**) represent positive signals (cell death), while blue fluorescence (**j**, **l**) represent nuclear staining. Scale bar = 50 μm. DMSO were used for reagents dissolution. The data are presented as the mean ± SD, *P < 0.05, **P < 0.01, *n* = 8.

AKI model was induced by cisplatin injection in wild type C57BL/6 mice. Pretreatment with Fer-1 significantly decreased the levels of sCr and BUN compared with cisplatin group (Fig. 1A, B). Tubular injury (widespread tubular cell death, severe cell shedding, interstitial edema, and brush border disruption in the kidney cortex) induced by cisplatin was also mitigated by Fer-1 as accessed by HE staining of the kidney tissues (Fig. 1C). In addition, Fer-1 remarkably reduced cell death induced by cisplatin measured by terminal deoxynucleotidyl transferase–mediated digoxigenin-deoxyuridine nick–end labeling (TUNEL) (Fig. 1D).

**Fig. 2:**
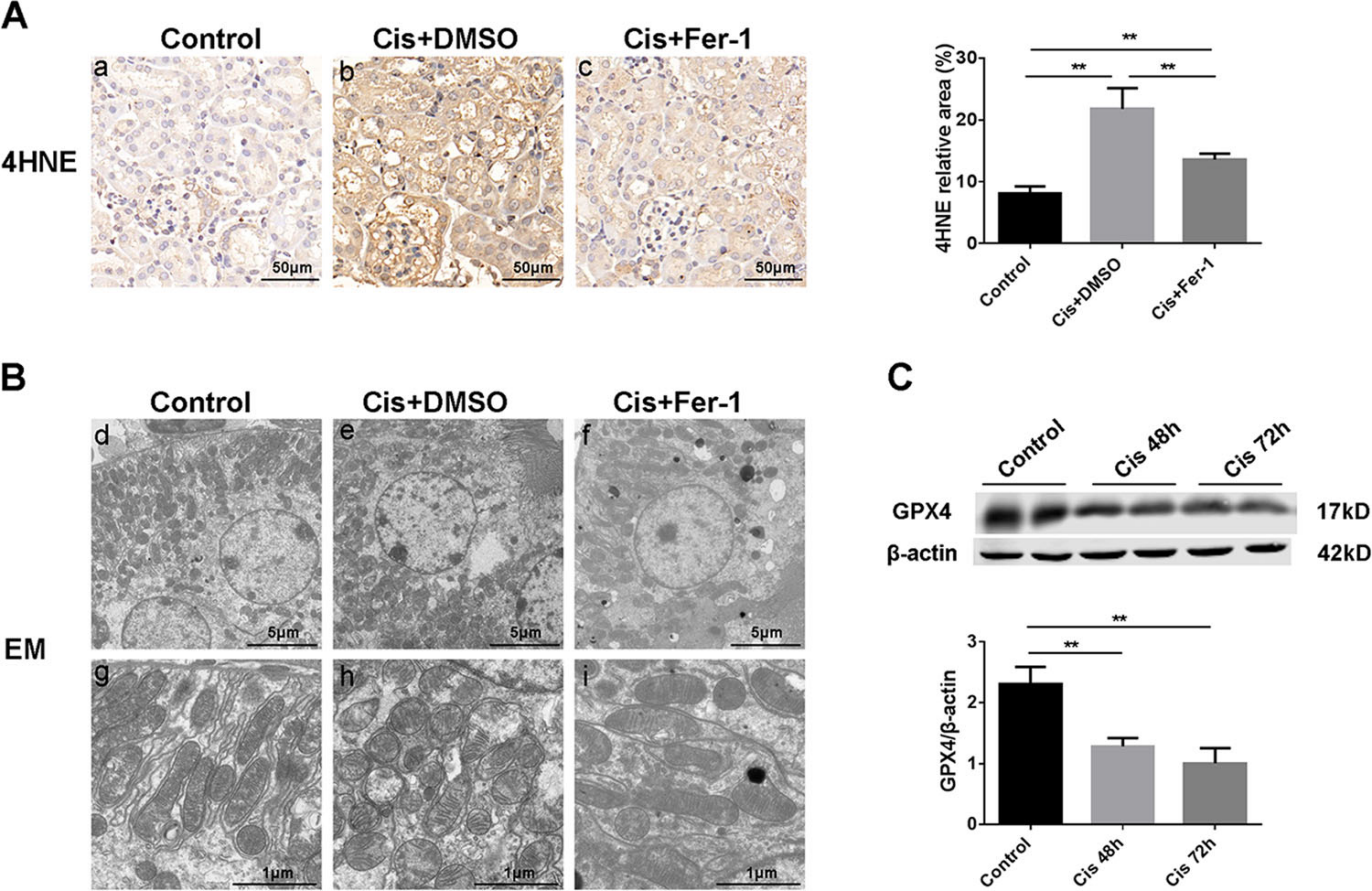
Renal lipid peroxidation and mitochondrial damage induced by cisplatin can be ameliorated by Fer-1. **A** Cortex expression of 4HNE determined by immunohistochemical staining after 48 h of indicated treatment, and the statistical analysis of the positive expression rate of each group. Scale bar = 50 μm. **B** Representative images of mitochondrial injury at 48 h under indicated treatment were observed by TEM. Scale bar = 5 μm in **d–f**. Scale bar = 1 μm in **g–i**. **C** Western blots analysis of GPX4 in renal tissue lysates from control group and cisplatin groups at 48 h and 72 h, respectively. Followed by statistical analysis of the ratio of optical density about GPX4 to β-actin in the above three groups. The data are presented as the mean ± SD, **P < 0.01, *n* = 8.

To further demonstrate the important role of ferroptosis in cis-AKI, the lipid peroxidation level (a key feature of ferroptosis) and ultrastructural changes were also determined. Immuohistochemical (IHC) staining of lipid peroxidation marker 4-hydroxynonenal (4HNE) levels showed that cisplatin could tremendously induce the production of 4-hydroxynonenal particularly in renal tubular cells, indicating an aberrant lipid peroxidation during cis-AKI. Moreover, Fer-1 pretreatment significantly decreased the expression of 4HNE (Fig. 2A). Electron microscopy observation showed that cisplatin treated kidney tissue exhibited more swollen mitochondria, a reduced number of cristae with a more lamellar phenotype compared with normal control. And these changes were significantly alleviated by Fer-1 pretreatment (Fig. 2B). Besides, we detected the protein level of glutathione peroxidase 4 (GPX4, a central regulator of ferroptosis) by western blot (Fig. 2C) and found a time-dependent decrease of GPX4 upon cisplatin stimulation, suggesting that cisplatin induced ferroptosis of tubular cell were related with GPX4 pathway. These data suggested that ferroptosis plays an essential role in cisplatin induced AKI.

### VDR agonist paricalcitol inhibited ferroptosis and protected cis-AKI

**Fig. 3:**
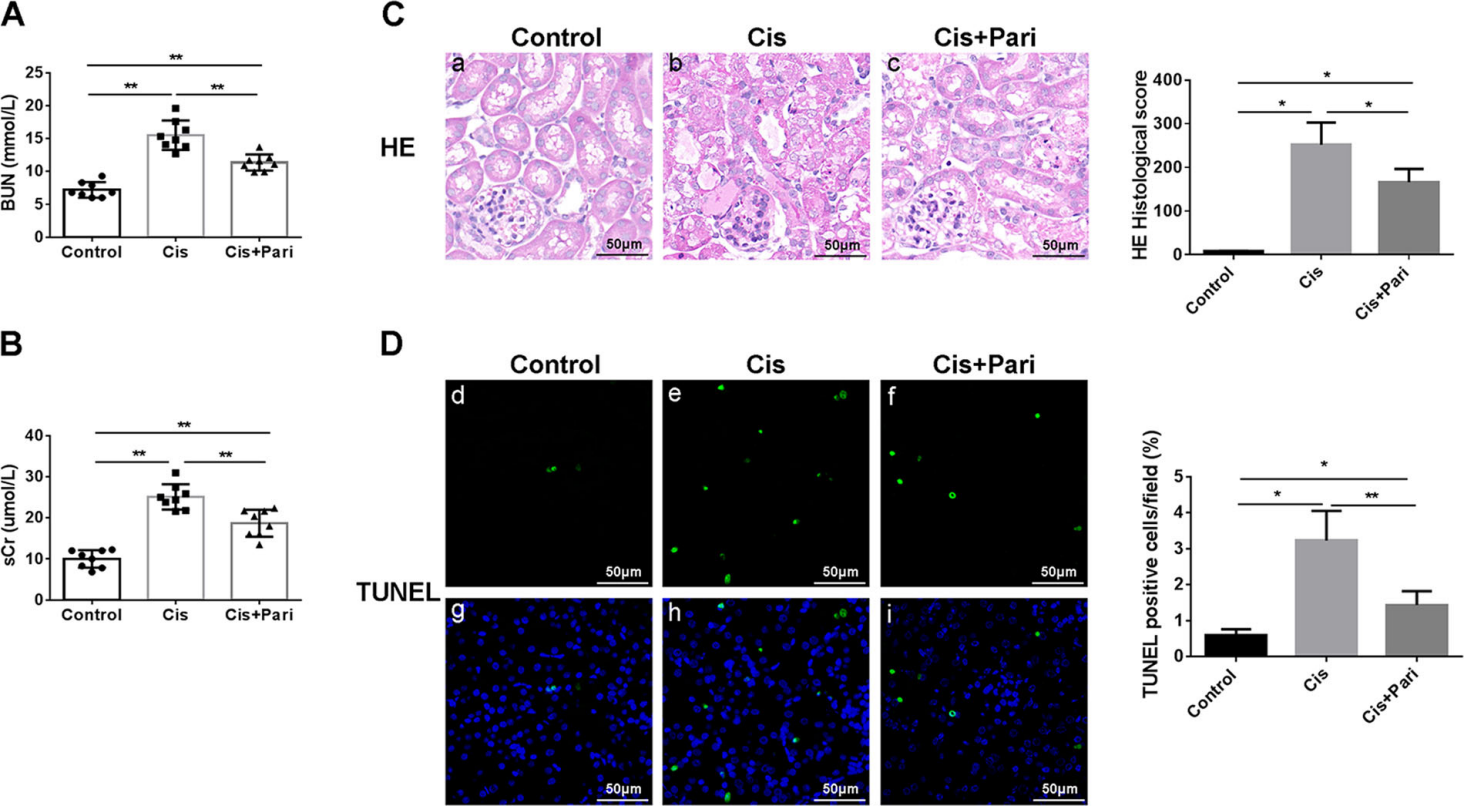
Kidney function loss and tissue injury induced by cisplatin were attenuated by paricalcitol pretreatment. **A**, **B** Serum BUN and Cr levels of each group at 48 h after indicative treatment were determined. **C** Histological changes of renal cortex by HE staining at 48 h after indicated treatment, and tissue injury scores in each group were statistically analyzed. Scale bar = 50 μm. **D** Cell death was assessed by TUNEL staining (green **d–f**). paricalcitol reduced TUNEL + cells in cisplatin induced AKI kidneys. blue fluorescence (**g–i**) represent nuclear staining. Representative image and quantification as mean ± SD of five mice per group. Scale bar = 50 μm. *P < 0.05, **P < 0.01, *n* = 8. Pari stands for VDR agonist paricalcitol.

**Fig. 4:**
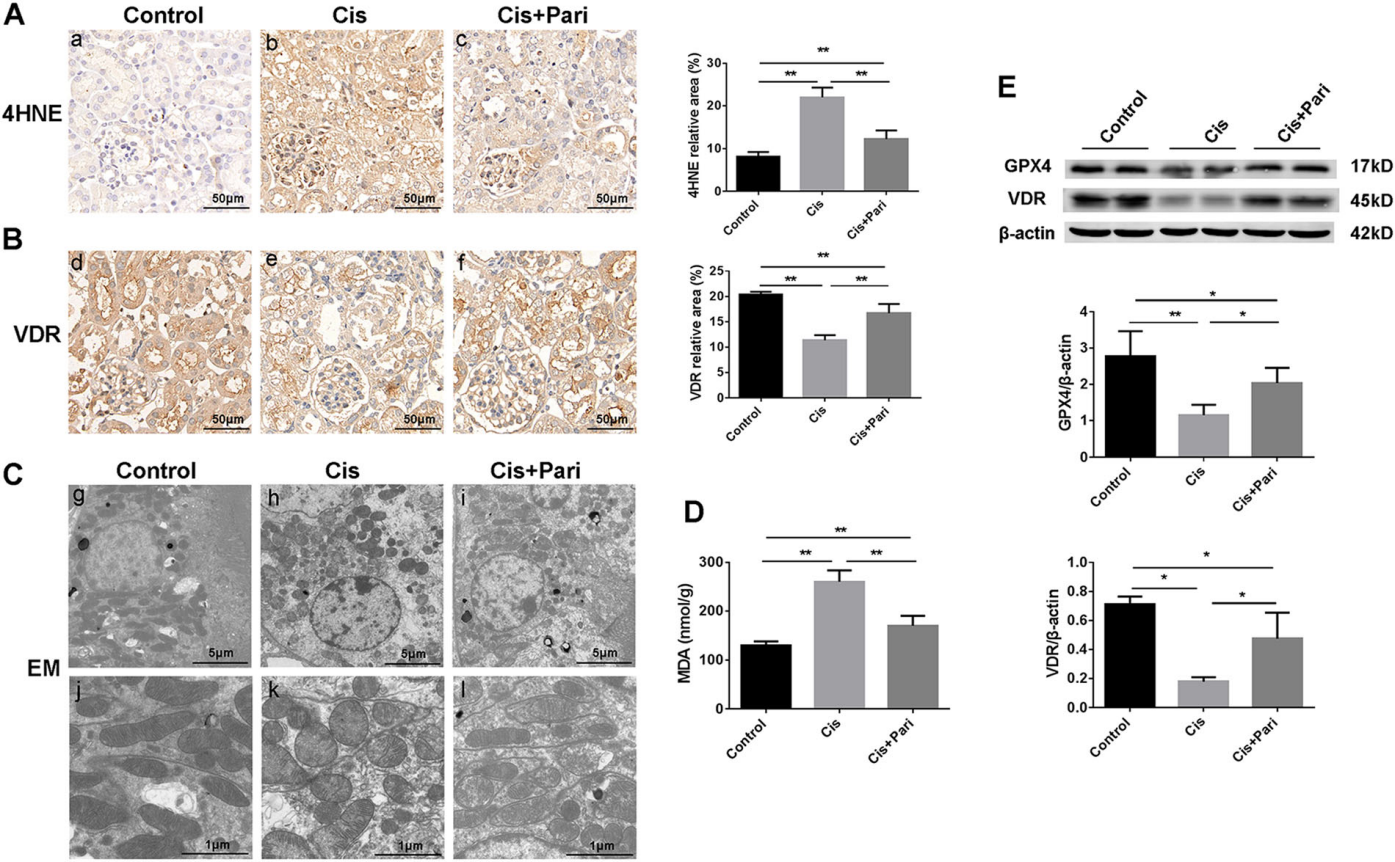
VDR agonist paricalcitol attenuated ferroptotic phenotype changes and mitochondrial injury induced by cisplatin. **A** Cortex expression of 4HNE determined by immunohistochemical staining after 48 h of indicated treatment, and the statistical analysis of the positive expression rate of each group (**a–c**). Scale bar = 50 μm. **B** The expression of VDR on renal tissue (**d–f**) were determined by immunohistochemical staining after 48 h of cisplatin with paricalcitol pretreatment, followed by the semi-quantitative statistical analysis of each group. Scale bar = 50 μm. **C** Representative images of mitochondrial injury at 48 h under indicated treatment were observed by TEM. Scale bar = 5 μm in **g–i**. Scale bar = 1 μm in **j–l**. **D** Quantitative analysis of the expression levels of MDA in kidney tissues of the three indicated groups. **E** Western blots analysis of GPX4 and VDR in renal tissue lysates from these three groups. The ratio of the optical density of GPX4 and VDR to β-actin were statistically analyzed. The data are presented as the mean ± SD, *P < 0.05, **P < 0.01, *n* = 8.

Vitamin D/VDR signaling has been widely reported to be protective in renal diseases like diabetic kidney disease and other chronic kidney diseases. Our recent work has found that VDR activation also exhibited protection against experimental AKI induced by lipopolysaccharide (LPS). Then we examined the effect of paricalcitol (a VDR agonist) in cis-AKI and cisplatin induced ferroptotic phenotype. Our data showed that paricalcitol pretreatment has significantly upregulated the expression of VDR and improved renal function (Fig. 3A, B) as assessed by plasma BUN and creatinine and reduced histologic injury (Fig. 3C) and cell death (Fig. 3D). Next, we found that paricalcitol could also decrease the accumulation of 4HNE (Fig. 4A) alone with restored expression of VDR (Fig. 4B) determined by immuohistochemical stainining. In addition, mitochondrial injury by cisplatin were also alleviated by VDR activation (Fig. 4C). In this part we also detected the production of malondialdehyde (MDA, a featured marker of lipid peroxidation) within the kidney tissue and the result showed that in line with the production of 4HNE, paricalcitol also decreased the accumulation of MDA induced by cisplatin (Fig. 4D). Surprisingly, our western blot data showed that paricalcitol could also restored the expression of GPX4 alone with VDR, of which both were decreased by cisplatin (Fig. 4E). These data indicate that VDR activation could also attenuate cis-AKI by decreasing ferroptotic cell death, probably associated with GPX4.

### VDR knock out downregulated GPX4 and aggravated cis-AKI

**Fig. 5:**
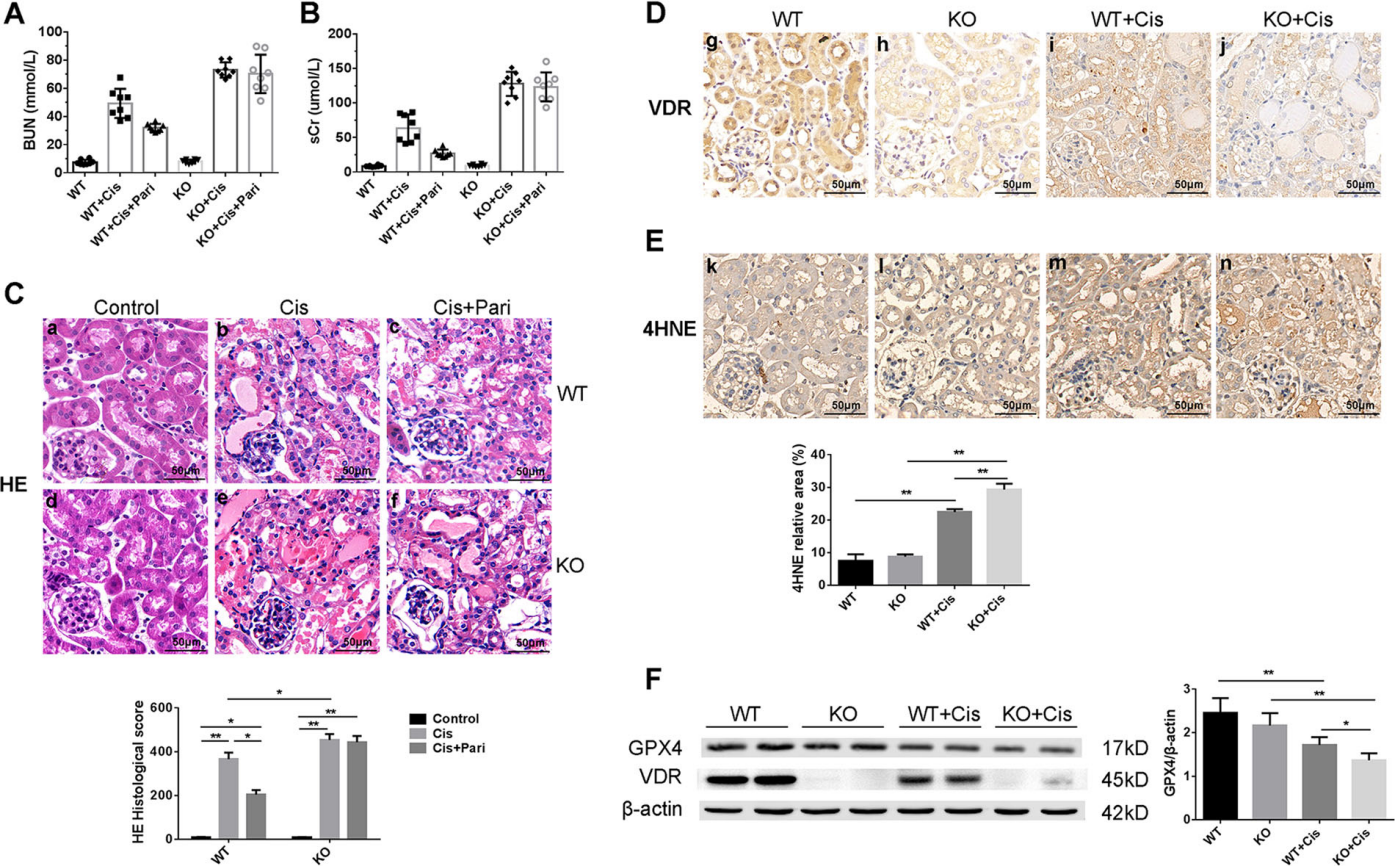
VDR knock out downregulated GPX4 and aggravate renal function loss, pathological changes, lipid peroxide peroxidation induced by cisplatin in mice model. **A**, **B** Serum BUN and Cr levels of six groups of wild type and transgenic VDR knockout mice at 72 h after cisplatin injection. **C** Histological changes of renal cortex by HE staining at 72 h after cisplatin injection, and tissue injury scores in each group were statistically analyzed. Scale bar = 50 μm. **D** Renal cortex expression of VDR determined by immunohistochemical staining in wild type and VDR-KO mice after 72 h of cisplatin treatment. Scale bar = 50 μm. **E** Cortex expression of 4HNE determined by immunohistochemical staining after 72 h treatment of cisplatin, followed by semi-quantitative statistical analysis of each group. Scale bar = 50 μm. **F** Western blots analysis of GPX4 and VDR in renal tissue lysates from these four groups. The ratio of the optical density of GPX4 to β-actin was statistically analyzed. The data are presented as the mean ± SD, *P< 0.05, **P < 0.01. *n* = 8.

On the basis of the observations that VDR activation may inhibit ferroptosis by affecting GPX4, we conducted the cis-AKI model with a VDR knockout mice. And not surprisingly, VDR deficiency exacerbated the renal function loss and tubular injury induced by cisplatin, and paricalcitol significantly decreased BUN and sCr, attenuated tissue injury in wild type mice, while in VDR-KO group, neither renal function nor tissue damage were affected (Fig. 5B, B, C). IHC staining showed the expression of VDR protein expression as well as location (Fig. 5D) in wild type mice and few expression in VDR-KO mice with or without cisplatin damage. 4HNE level within kidney tissue were much higher in VDR knockout mice than wild type upon cisplatin toxicity (Fig. 5E). More importantly we found that VDR deficiency resulted in a decreased expression of GPX4 in kidney tissue of VDR-KO mice without cisplatin injection and a much more serious decrease under cisplatin toxicity by western blot (Fig. 5F). In cell culture experiments we also found that cisplatin induced much severer cell death in VDK deficient HK-2 cells than wild type cells assessed by PI and Hoechst staining (Fig. 8A). Consistent with the in vivo data, VDR deficiency in HK-2 cell also decreased the protein expression of GPX4 compared with control cells, and an augmented decrease was detected after cisplatin damage (Fig. 8B). In this regard, a much closer relationship between VDR and GPX4 as well as ferroptosis can be concluded.

### GPX4 are transcriptionally regulated by transcriptional factor VDR

**Fig. 6:**
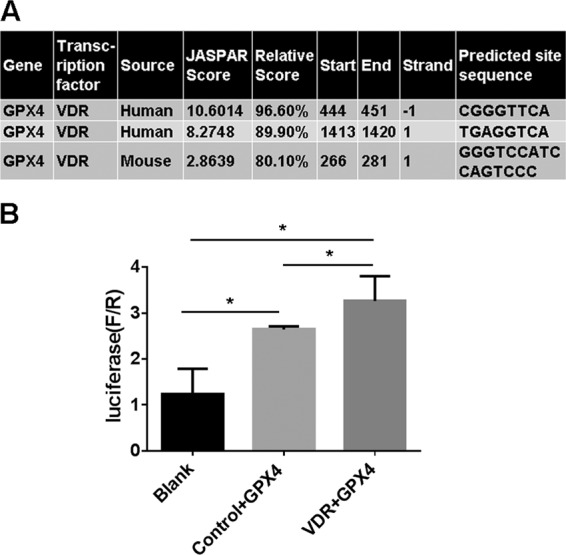
GPX4 are transcriptionally regulated by transcriptional factor VDR. **A** Predicted VDR binding sites in human and mouse GPX4 gene promoter regions. Human and mouse GPX4 gene upstream regions were analyzed for VDR binding sites by JASPAR online analysis (http://jaspar.genereg.net). The potential binding sites of 90% chance (relative score) to bind VDR are listed. **B** Luciferase activity assay were conducted to assess the interaction between GPX4 gene promoter regions and transcription factor VDR in human 293 T cells. Luciferase reporter plasmid containing GPX4 promoter sequence (or blank sequence) were transinfected into the control 293 cells or VDR expressed cells, luciferase activity were quantitatively analyzed. The data are presented as the mean ± SD, *P < 0.05, **P < 0.01. *n* = 3.

To further investigate the mechanism of the regulation of VDR on GPX4, we went on to explore the inherent relation between VDR and GPX4, as VDR is a nuclear transcription regulator. We propose that GPX4 might be directly trans-regulated by VDR, by Using the JASPAR database (http://jaspar.genereg.net), we analyzed the promoter sequence of GPX4 from both human and mice homologous sequence. The analysis identified three putative VDR binding sites for mice and human (Fig. 6A). These bioinformatics predictions strongly indicate a transcriptional regulation of VDR upon GPX4. Subsequently we examined the binding of VDR to these sites by luciferase reporter gene assay. When the luciferase reporter plasmid containing GPX4 promoter sequence were transinfected into the control 293 cells (with VDR expressed regularly), abundant luciferase signal were detected, while significantly enhanced luciferase activity were obtained when transinfected into the VDR overexpressed 293 cells (Fig. 6b). Supporting the direct binding of VDR to GPX4 promoter, indicating a transregulational effect of VDR on GPX4.

### GPX4 deficiency eliminated the protective effect of VDR

**Fig. 7:**
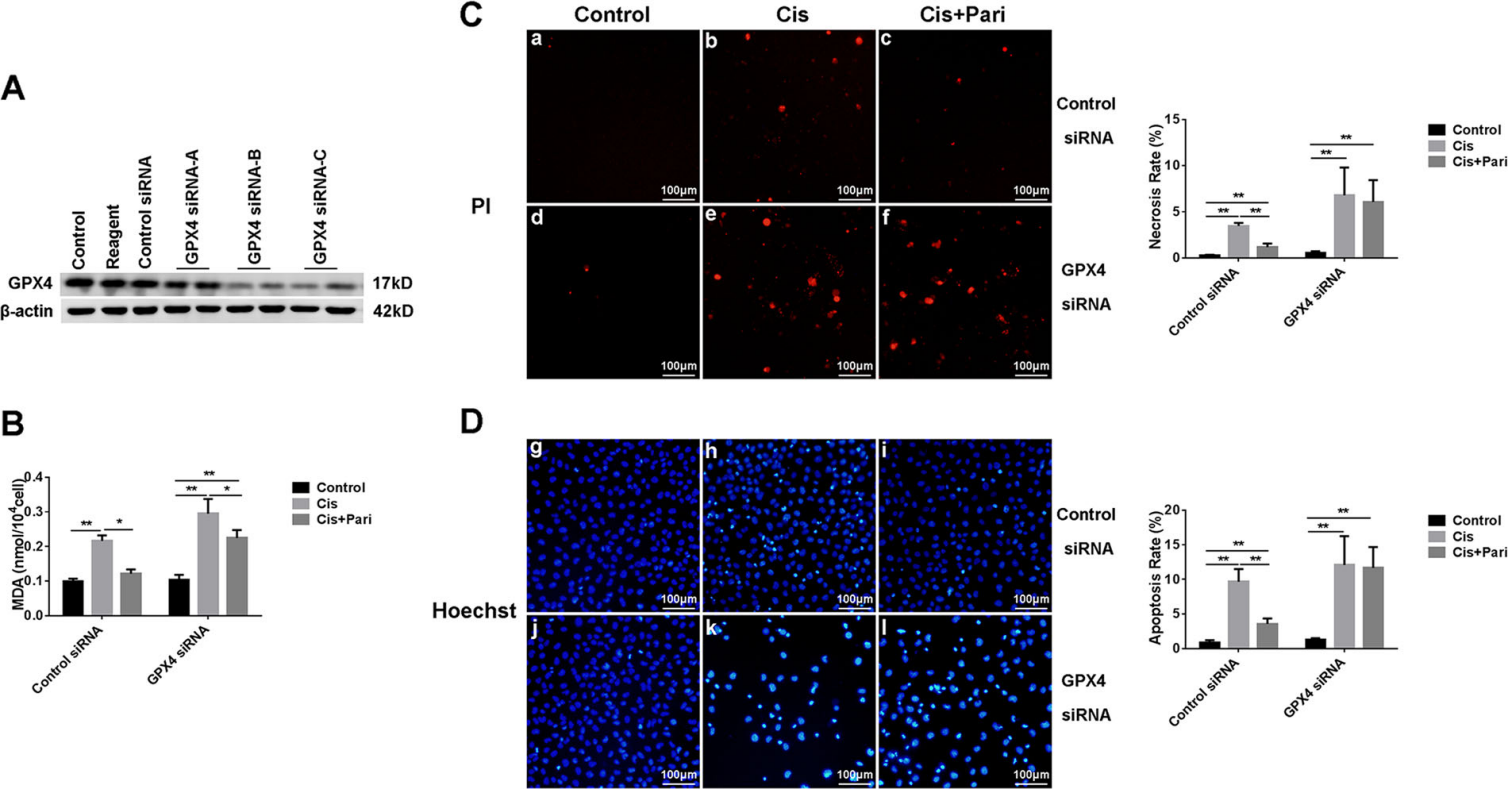
The protective effect of paricalcitol on cisplatin induced damage was reduced after GPX4 inhibition by siRNA in HK-2 cells. **A** Western blots analysis of GPX4 protein in HK-2 cells after transfection with transfection reagent, control siRNA or 3 alternative GPX4 siRNAs(GPX4 siRNA A/B/C), respectively. **B** Quantitive analysis of MDA levels in HK-2 cells in different conditions. **C** Necrotic cell death evaluation by PI staining(red) and statistical analysis of necrosis rate in each group of cells. The cells in panels **a–c** were transfected with control siRNA, while the cells in panels **d–f** were transfected with GPX4 siRNA. Scale bar = 100 μm. **D** Hoechst staining and statistical analysis of apoptosis rates in each group. Blue fluorescence is the staining of the nucleus, where the brighter blue shows the apoptotic cell nucleus. cells in panels **g–i** were transfected with control siRNA, while the cells in panels **j–l** were transfected with GPX4 siRNA. Scale bar = 100 μm. The data are presented as the mean ± SD, *P < 0.05, **P < 0.01. *n* = 3.

To further demonstrate the ferroptosis-inhibiting effect of VDR as well as its cytoprotective potential were dependent on regulation of GPX4. We conducted in vitro experiment with HK-2 cells. After inhibiting the expression of GPX4 by siRNA (Fig. 7A), cisplatin remarkably induced more MDA production (Fig. 7B) and much more cell death including both necrotic and apoptotic cells (Fig. 7C and D). In addition, pretreatment of VDR agonist paricalcitol has significantly reduced the production of MDA as well as cell death in control cells, however, this effect was largely abolished in the GPX4 deficient cell. These data confirmed that the protective effect of VDR activation were dependent of GPX4, at least partly.

### Paricalcitol alleviated erastin induced cell death in HK-2 cells

**Fig. 8:**
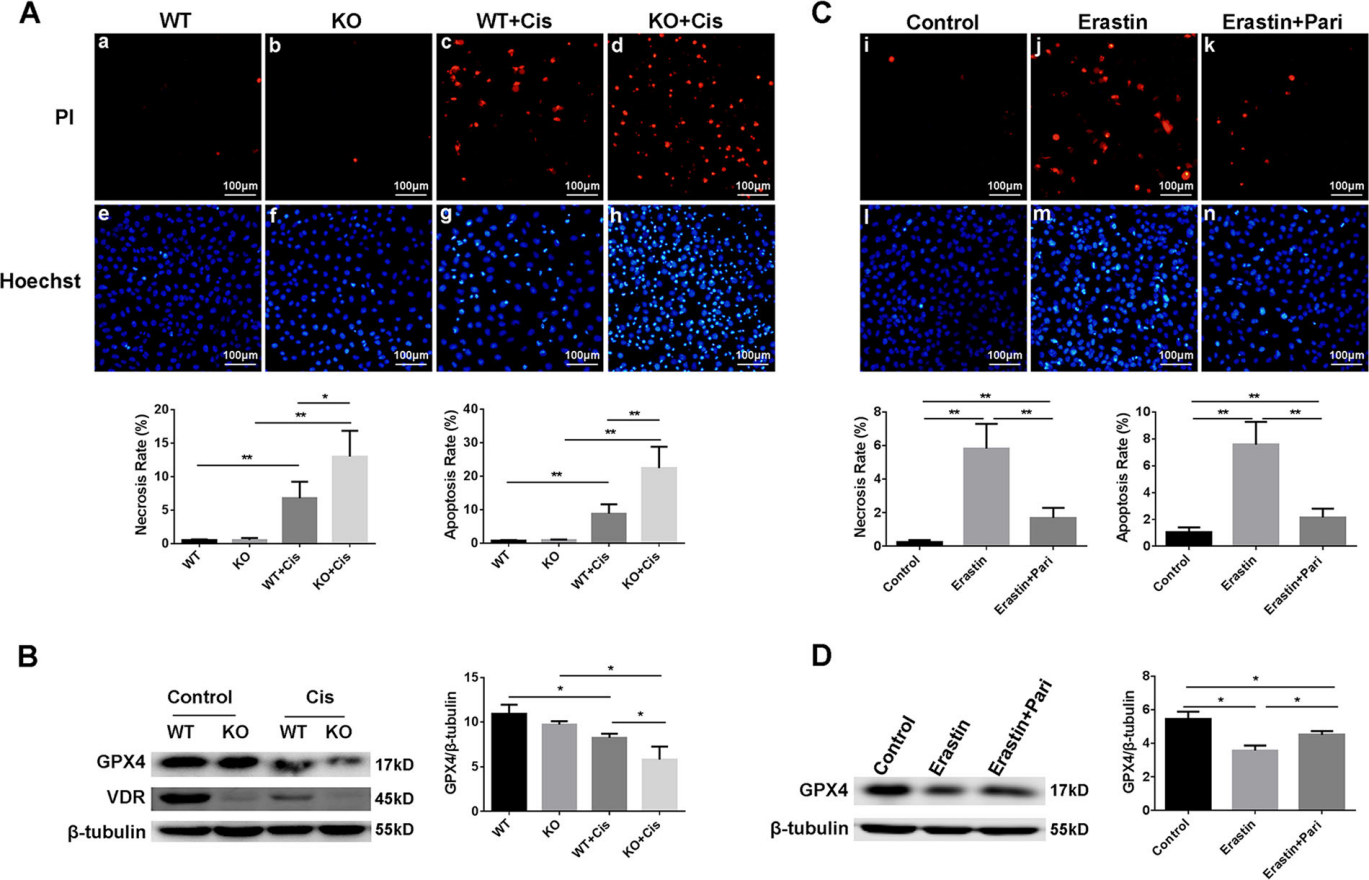
Worsened cell injury in VDR deficient HK-2 cell induced by cisplatin (A and B), Paricalcitol alleviated Erastin induced cell death in HK-2 cells (C and D). **A** PI (**a–d**) and Hoechst (**e–h**) staining of wild type and VDR-KO cells after cisplatin treated, and statistical analysis of necrosis and apoptosis rates respectively of each group of cells. Scale bar = 100 μm. **B** Western blots analysis of GPX4 and VDR in cells from these four groups. The ratio of the optical density of GPX4 to β-tubulin was statistically analyzed. **C** PI (**i–k**) and Hoechst (**l–n**) staining of cells from control, erastin, erastin + paricalcitol groups, respectively followed with statistical analysis of necrosis and apoptosis rates of each group of cells. Scale bar = 100 μm. **D** Western blots analysis of GPX4 in cells from these three groups. The ratio of the optical density of GPX4 to β-tubulin was statistically analyzed. The data are presented as the mean ± SD, *P < 0.05, **P < 0.01. *n* = 3.

Since we have observed the protective effect of paricalcitol as well as the regulation of VDR on ferroptosis in cisplatin induced injury,we further verified these effects of paricalcitol in Erastin (a specific inducer of ferroptosis) induced cell death in HK-2 cells. Our data showed that Erastin (10 μM) could induce remarkable cell death assessed by PI and Hoechest staning (Fig. 8C), and pretreatment with paricalcitol could significantly alleviate erastin induced cell death in HK-2 cells. Western blot data showed that paricalcitol could also restore GPX4 protein expression downregulated by erastin induced injury (Fig. 8D). These data further supported the potential regulation of vitamin D/VDR in ferroptosis in renal tubular cells.

## Discussion

Cisplatin is one of the most widely used chemotherapeutic agents, and AKI is the main risk of cisplatin-based therapy^2^. Apoptosis were generally believed to be the key pathological process and cell death form in cisplatin induced AKI. It is regulated by Bax and P53 and other relevant factors like miRNAs and Histone deacetylases^14–16^. Ramesh G et al. have reported that cisplatin could induce both apoptosis and necrosis which is mediated by TNF receptor 2 (TNFR2) in mice AKI model^17^. In recent years, researches have showed that ferroptosis is playing an emerging role in clinical AKI as well as rodent models induced by IRI and rhabdomyolysis^18,19^. Earlier before two decades, Baliga et al.’s work has suggested the importance of ferric ion in pathological processes of cisplatin induced renal injury in mice^8^. At that time ferroptosis had not been recognized and defined then. While, in our present work we have observed an obvious ferroptotic phenotype in cisplatin induced AKI mice model. Cisplatin injection could remarkably increase lipid peroxidation and ROS production, which were prevented by ferroptosis inhibitor Fer-1 accompanied with improved renal function and tissue injury. Even through it is not the only type of cell death processes, our work is consistent with previous report and showed that ferroptosis plays important role in cisplatin induced AKI^8,20^. More importantly, our data has showed that cisplatin has obviously decreased the expression of GPX4 in tubular cells, which suggest that cisplatin induced ferroptosis in renal cells may be mediated through GPX4 pathway.

The protective effect of vitamin D/VDR in kidney diseases has been validated by a lot of research works including ours^10,21^. While most of those work mainly focused on chronic kidney diseases like diabetic kidney disease. Quite few of them have explored the effect of VDR in clinical AKI or in vivo models. Azak A et al. has reported that VDR activator paricalcitol could mitigate renal ischemia/reperfusion injury in rats^22^. Our quite recent work also showed a promising protection of vitamin D-VDR signaling against apoptosis in an AKI model induced by LPS^23^. In another way, supplementation of Vitamin D3 has also been reported to up-regulate VDR and decrease reduces cellular necrosis in lung tissue of mice infected by tuberculosis^24^. Our present work has showed that VDR activation by paricalcitol could significantly improve renal function and attenuate tissue injury in cisplatin induced AKI model in mice. By further investigation we found that VDR activation reduced the lipid peroxidation level and alleviate mitochondrial injury, more importantly, restored GPX4 expression loss, the main feature of ferroptosis. Indicating a mechanism of the inhibitory effect of VDR activation on ferroptosis in cisplatin induced AKI. We consequently verified this regulation mechanism using an VDR-KO mice model as well as cell culture which resulted in an aggravated ferroptotic phenotype and renal injury, meanwhile further downregulated expression of GPX4. So far, the protective effect of VDR activation were not reported in cisplatin induced AKI. In Hamzawy et al.’s work^25^, 22-oxacalcitriol, another VDR agonist were also reported to be renoprotective against IRI induced AKI by inhibiting apoptosis and enhancement of autophagy. Our data has suggested that VDR activation could alleviate renal injury by inhibiting ferroptosis. Cell culture work using Erastin, a specific inducer of ferroptosis, further supported the potential regulation of VDR on ferroptosis. We have also observed a relationship between VDR and GPX4, a central regulator of ferroptosis. Bioinformatic analysis and further observation demonstrated that GPX4 could be trans-regulated by VDR, which suggested that the regulatory effect of VDR on ferroptosis are dependent on GPX4. On the other hand VDR activation or vitamin D administration were reported to influences NADPH Oxidase (NOX2) Activity^26^, protects against mitochondrial dysfunction, and cell apoptosis^27^, in addition, vitamin D could prevent high glucose induced oxidative stress of renal tubular cells^28^. Moreover, VDR inhibition induced lipid metabolism abnormality, reduced eNOS and ApoE levels, promoted lipid peroxidation^29^. These works might have indirectly indicated a potential relation between vitamin D/VDR signaling and ferroptosis. However, the effect of VDR activation on ferroptosis have not been reported yet. And our work has found that VDR activation is cytoprotective via inhibiting ferroptosis, and this effect was mediated by regulating GPX4. It should be noted that, in the kidney tissue of VDR-KO mice without cisplatin injection, the expression of GPX4 were only slightly downregulated which was not in line with the downregulation of VDR. While upon a cisplatin treatment the expression of GPX4 were tremendously decreased compared with wide type mice. Alone with the luciferase reporter gene assay results, these data has supported a trans-regulatory effect of VDR on GPX4, more precisely to say, VDR may not be the key regulator of GPX4, however, the overall effect of VDR on ferroptosis inhibition and renal protection is obviously there, and we speculate that VDR might also affect ferroptosis through other mechanisms and of course VDR activation could also exert its renal protective effect via regulation of other cell death processes as mentioned before. Another question like the exact mechanism of how cisplatin triggered ferroptosis still warrant further future research works.

In summary, our present work has confirmed the important role of ferroptosis in cisplatin induced AKI, while VDR activation could attenuate cisplatin induced AKI by inhibiting ferroptosis, and this protective effect was mediated by trans-regulation of GPX4. Our work suggested that ferroptosis could be a promising target for AKI prevention and treatment, while VDR activation might be a potential strategy for that.

## Materials and methods

### Reagents

Cisplatin was provided by the Department of Oncology, Third Xiangya Hospital, Central South University. Paricalcitol was provided by professor Yanchun Li’s laboratory at the University of Chicago. Ferrostatin-1 (Fer-1) and Erastin were purchased from APExBIO (USA). Anti-GPX4 rabbit monoclonal antibody (ab125066), anti-4HNE rabbit polyclonal antibody (ab46545), and anti-VDR rabbit monoclonal antibody (ab109234) were purchased from Abcam (UK). Anti-β-actin rabbit monoclonal antibody (CST4967) and anti-β-tubulin (9F3) rabbit monoclonal antibody (CST2128) were purchased from CST (USA). Secondary antibodies were IRDye 800CW Goat anti-Rabbit IgG, purchased from Licor (USA). The BUN(Urea Assay Kit) and sCr(Creatinine Assay kit (sarcosine oxidase)) test kits were obtained from Nanjing Jiancheng Bioengineering Institute (Nanjing, China). The TUNEL cell death detection kit (cat. no. 40306ES50) was purchased from Yeasen (Shanghai, China). Hoechst 33342 and Propidium Iodide (PI) staining solutions as well as the malondialdehyde (MDA) detecting kit were purchased from Solarbio.Co. (Beijing, China). Transfection reagent riboFECTTM CP and siRNAs that specifically inhibit GPX4 were provided by Ribobio (Guangzhou, China).

### Animals and experimental protocol

A total of 72 male C57BL/6 mice were purchased from Slyke jingda Biotechnology Company (Certificate SCXK2016-0002; Hunan, China). All of them were fed under SPF-condition. They were randomly divided into five groups: Control group (*n* = 8), Cisplatin (20 mg/kg dissolved in saline) only group (*n* = 16), Cisplatin + paricalcitol (0.2ug/kg dissolved in sterile water for injection and 20% propylene glycol) group (*n* = 16), Cisplatin + DMSO group (*n* = 16), Cisplatin + Fer-1 (5 mg/kg dissolved in DMSO) group (*n* = 16), were administered intraperitoneally. Cisplatin was injected once to mice, while Fer-1 was injected once an hour before cisplatin, and paricalcitol was injected once daily for five consecutive days before cisplatin. Each eight mice were sacrificed at 48 h and 72 h, respectively after cisplatin injection, and eight mice in the control group were sacrificed together with mice at 72 h. The serum and kidney tissues of the mice were collected.

VDR knockout transgenic mice were initially purchased from Nanjing Biomedical Research Institute of Nanjing University and bred in our experimental animal center of Central South University, wild type (WT) or knockout (KO) mice were respectively separated into two groups: Control group, Cisplatin group, with eight mice in each group. Cisplatin administration was conducted as the above. All mice were sacrificed 72 h after cisplatin treatment.

### Cell culture and treatment

Human proximal tubular epithelial cells (HK-2 cells) and their VDR knockout (VDR-KO) cell lines were provided by the Institute of Kidney disease, Central South University. The HK-2 cells were divided into blank control group, control siRNA group, and GPX4 siRNA group (3 alternative siRNAs against GPX4 mRNA named as GPX4 siRNA-A, GPX4 siRNA-B, GPX4 siRNA-C were used to inhibit GPX4). The cells were seeded at 5 × 10^4^ cells/well in six well plates. After 24 h incubation at 37 °C, the cells were transfected with 50 nM siRNA. The siRNA was mixed with the serum-free medium and transfection reagents according to the manufacturer’s instructions. After 6 h, the cells were replaced with complete medium containing serum and penicillin-streptomycin solution. Cellular protein was extracted after 48 h of culture, and transfection efficiency was evaluated by western blot assay. The most efficient siRNA silencing GPX4(GPX4 siRNA-B) was used for the subsequent experiments.

Then the cells were divided into six groups: control siRNA group, control siRNA + cisplatin group, control siRNA + cisplatin + paricalcitol group, GPX4 siRNA group, GPX4 siRNA + cisplatin group, GPX4 siRNA + cisplatin + paricalcitol group. The transfection step was the same as above, and the paricalcitol was added together with the complete medium at the time of transfection (400 nM). After pretreatment with paricalcitol for 24 h, cisplatin (20 ug/ml) was added, followed by PI/Hoechst staining or MDA level detection after another 24 h.

The HK-2 cells were divided into three groups: control group, erastin group, and erastin + paricalcitol group. Paricalcitol (400 nM) was added 24 h after the cells were seeded, and the erastin (10 μM) was added 24 h after paricalcitol, followed by Hoechst/PI staining or GPX4 protein detection after another 24 h.

The HK-2 cells and VDR-KO cells were respectively divided into two groups: control group and cisplatin group. 48 h after seeded, the cells were treated by cisplatin (20 ug/ml), another 24 h later, they were collected for PI/Hoechst staining or GPX4 protein detection.

### Measurement of BUN, sCr, MDA

Renal tissues and blood were collected for biochemical analysis. BUN and sCr levels in serum and MDA levels in renal tissues were measured using the corresponding detection kits in accordance with the manufacturer’s instructions. In addition, lysate of HK-2 cells were also collected for detection of MDA levels.

### Mitochondrial morphology observation by electron microscopy

Briefly, 1 mm^3^ fresh renal cortex was removed and quickly placed in an electron microscopy fixative at 4 °C. Tissues were embedded and cut into 60–80 nm ultrathin sections and then subjected to uranium lead double staining. Transmission electron microscopy was used to observe renal tubular epithelial cells and image acquisition.

### Renal tissue histopathological, immunohistochemistry (IHC), and TUNEL assessment

Fresh renal tissues were collected, fixed immediately with formalin, and then embedded in paraffin, followed cut into a thickness of 5 μm sections, which were used for hematoxylin-eosin (H&E) staining, 4HNE, or VDR immunohistochemistry, or TUNEL fluorescent staining. The degree of renal interstitial damage in mice is assessed based on the tubular endothelial cell morphology, brush border integrity, number of renal epithelial casts, and lumen necrotic cells by the method as previously reported^30^. The intensities of 4HNE and VDR in the photos were detected by Image J software. Cell death rate was quantified.

### Western blot analysis

Briefly, after tissue proteins were extracted, proteins of each group were separated by a 12% SDS-polyacrylamide gel and transferred onto a polyvinylidene difluoride membrane. The membrane was blocked with 0.1% (w/v) BSA solution on a shaker for 2 h. Then, the membrane was incubated in the corresponding primary antibody at 4 °C for one night, followed in the fluorescent secondary antibody for 1 h. Finally, the membranes were visualized by the Image Studio software and band intensities were quantified using Image J gel analysis software. All experiments were repeated at least three times.

### Hoechst and PI staining of cells

Hoechst and PI stains were formulated into working fluids and added to cells that had been treated accordingly. The cells were observed and photographed under a fluorescence microscope after incubation at 37 °C for 5 min. Quantification of apoptotic cells or necrotic cells were performed by taking the photos in random fields and counting at least 200 cells in five random fields of each well.

### Statistical analysis

All data values referenced above were presented as means ± SD. Statistical comparisons were carried out using unpaired two-tailed Student’s *t* test or one-way analysis of variance (ANOVA) as appropriate. Statistical significance was defined as *p* < 0.05.

## References

[1] Bellomo R, Kellum JA, Ronco C (2012). Acute kidney injury. Lancet.

[2] Ozkok A, Edelstein CL (2014). Pathophysiology of cisplatin-induced acute kidney injury. Biomed. Res. Int.

[3] Xu Y (2015). A role for tubular necroptosis in cisplatin-induced AKI. J. Am. Soc. Nephrol..

[4] Tristao VR (2012). Nec-1 protects against nonapoptotic cell death in cisplatin-induced kidney injury. Ren. Fail.

[5] Herzog C, Yang C, Holmes A, Kaushal GP (2012). zVAD-fmk prevents cisplatin-induced cleavage of autophagy proteins but impairs autophagic flux and worsens renal function. Am. J. Physiol. Ren. Physiol..

[6] Dixon SJ (2012). Ferroptosis: an iron-dependent form of nonapoptotic cell death. Cell.

[7] Stockwell BR (2017). Ferroptosis: a regulated cell death nexus linking metabolism, redox biology, and disease. Cell.

[8] Baliga R, Zhang Z, Baliga M, Ueda N, Shah SV (1998). In vitro and in vivo evidence suggesting a role for iron in cisplatin-induced nephrotoxicity. Kidney Int.

[9] Pike JW, Meyer MB, Lee SM, Onal M, Benkusky NA (2017). The vitamin D receptor: contemporary genomic approaches reveal new basic and translational insights. J. Clin. Invest.

[10] Mansouri L (2017). Vitamin D receptor activation reduces inflammatory cytokines and plasma MicroRNAs in moderate chronic kidney disease—a randomized trial. BMC Nephrol..

[11] Wang Y (2012). Vitamin D receptor signaling in podocytes protects against diabetic nephropathy. J. Am. Soc. Nephrol..

[12] Zhang W (2017). 1,25-(OH)2D3 and its analogue BXL-628 inhibit high glucose-induced activation of RhoA/ROCK pathway in HK-2 cells. Exp. Ther. Med.

[13] Yi B (2016). Vitamin D receptor down-regulation is associated with severity of albuminuria in type 2 diabetes patients. J. Clin. Endocrinol. Metab..

[14] Wei Q, Dong G, Franklin J, Dong Z (2007). The pathological role of Bax in cisplatin nephrotoxicity. Kidney Int.

[15] Liu, J. (2018). Histone deacetylase inhibitors protect against cisplatin-induced acute kidney injury by activating autophagy in proximal tubular cells. Cell Death Dis..

[16] Du B (2017). MiR-30c regulates cisplatin-induced apoptosis of renal tubular epithelial cells by targeting Bnip3L and Hspa5. Cell Death Dis..

[17] Ramesh G, Reeves WB (2003). TNFR2-mediated apoptosis and necrosis in cisplatin-induced acute renal failure. Am. J. Physiol. Ren. Physiol..

[18] Linkermann A (2014). Synchronized renal tubular cell death involves ferroptosis. Proc. Natl. Acad. Sci. USA.

[19] Guerrero-Hue M (2019). Curcumin reduces renal damage associated with rhabdomyolysis by decreasing ferroptosis-mediated cell death. FASEB J..

[20] Liu H, Baliga R (2003). Cytochrome P450 2E1 null mice provide novel protection against cisplatin-induced nephrotoxicity and apoptosis. Kidney Int.

[21] Yang S (2018). Vitamin D receptor: a novel therapeutic target for kidney diseases. Curr. Med. Chem..

[22] Azak A (2013). Effect of novel vitamin D receptor activator paricalcitol on renal ischaemia/reperfusion injury in rats. Ann. R. Coll. Surg. Engl..

[23] Du J (2019). Vitamin D receptor activation protects against lipopolysaccharide-induced acute kidney injury through suppression of tubular cell apoptosis. Am. J. Physiol. Ren. Physiol..

[24] Wahyunitisari MR, Mertaniasih NM, Amin M, Artama WT, Koendhori EB (2017). Vitamin D, cell death pathways, and tuberculosis. Int. J. Mycobacteriol.

[25] Hamzawy M (2019). 22-oxacalcitriol prevents acute kidney injury via inhibition of apoptosis and enhancement of autophagy. Clin. Exp. Nephrol..

[26] Cui C (2017). Vitamin D receptor activation influences NADPH Oxidase (NOX2) Activity and protects against neurological deficits and apoptosis in a rat model of traumatic brain injury. Oxid. Med. Cell Longev..

[27] Chen C, Luo Y, Su Y, Teng L (2019). The vitamin D receptor (VDR) protects pancreatic beta cells against Forkhead box class O1 (FOXO1)-induced mitochondrial dysfunction and cell apoptosis. Biomed. Pharmacother..

[28] Zhu X, Wu S, Guo H (2019). Active Vitamin D and Vitamin D receptor help prevent high glucose induced oxidative stress of renal tubular cells via AKT/UCP2 signaling pathway. Biomed. Res. Int.

[29] Ding Y, Liao W, Yi Z, Xiang W, He X (2015). Cardioprotective role of vitamin D receptor in circulating endothelial cells of ApoE-deficient mice. Int J. Clin. Exp. Med.

[30] Paller MS, Hoidal JR, Ferris TF (1984). Oxygen free radicals in ischemic acute renal failure in the rat. J. Clin. Invest.

